# Pleiotropic effects of RsmA and RsmE proteins in *Pseudomonas fluorescens* 2P24

**DOI:** 10.1186/s12866-020-01880-x

**Published:** 2020-07-02

**Authors:** Yang Zhang, Bo Zhang, Haiyan Wu, Xiaogang Wu, Qing Yan, Li-Qun Zhang

**Affiliations:** 1 College of Agriculture, Guangxi University, Nanning, 530004 China; 2 Department of Plant Sciences and Plant Pathology, Montana State University, Bozeman, 59717 USA; 3 College of Plant Protection, China Agricultural University, Beijing, 100193 China

**Keywords:** *Pseudomonas fluorescens*, RsmA/RsmE, 2,4-DAPG, Biofilm, Motility

## Abstract

**Background:**

*Pseudomonas fluorescens* 2P24 is a rhizosphere bacterium that produces 2,4-diacetyphloroglucinol (2,4-DAPG) as the decisive secondary metabolite to suppress soilborne plant diseases. The biosynthesis of 2,4-DAPG is strictly regulated by the RsmA family proteins RsmA and RsmE. However, mutation of both of *rsmA* and *rsmE* genes results in reduced bacterial growth.

**Results:**

In this study, we showed that overproduction of 2,4-DAPG in the *rsmA rsmE* double mutant influenced the growth of strain 2P24. This delay of growth could be partially reversal when the *phlD* gene was deleted or overexpression of the *phlG* gene encoding the 2,4-DAPG hydrolase in the *rsmA rsmE* double mutant. RNA-seq analysis of the *rsmA rsmE* double mutant revealed that a substantial portion of the *P. fluorescens* genome was regulated by RsmA family proteins. These genes are involved in the regulation of 2,4-DAPG production, cell motility, carbon metabolism, and type six secretion system.

**Conclusions:**

These results suggest that RsmA and RsmE are the important regulators of genes involved in the plant-associated strain 2P24 ecologic fitness and operate a sophisticated mechanism for fine-tuning the concentration of 2,4-DAPG in the cells.

## Background

Bacteria use a complex interconnecting mechanism to recognize and adapt to changes in their environment and reprogram numerous cellular processes in response to physiological homeostasis. An important element in this complex regulatory network is the Gac/Rsm cascade pathway [1]. This pathway is conserved in numerous Gram-negative bacteria and mediates the post-transcriptional regulation of diverse genes required for bacterial virulence and metabolism [2]. These include genes for the expression of extracellular enzymes [3], carbon storage compounds [4], motility [5], the formation of biofilm [6], and the production of secondary metabolites and virulence factors [7, 8]. Signal transduction initially involves the GacS/GacA two-component system which consists of the histidine kinase protein GacS and its cognate response regulator GacA. Upon interaction with unknown signals, the membrane sensor GacS autophosphorylates and activates the GacA by phosphorylation. Phosphorylated GacA positively controls transcription initiation of one or more small non-coding RNAs (sRNA) genes, depending on the bacterial species, such as *rsmX*, *rsmY* and *rsmZ* in *Pseudomonas protegens* CHA0 [9]. A conserved upstream activating sequence (UAS) is found to be necessary for GacA protein to activate the expression of these sRNAs [10]. These sRNAs exhibit high affinity for the CsrA/RsmA family protein. The CsrA/RsmA family protein can inhibit translation or stability of transcripts of its target genes by binding to sites overlapping the SD sequence or ribosome binding sites of target mRNAs, thus influencing ribosome access [11]. In addition, varying numbers of RsmA orthologs have been described in different bacteria and these proteins exhibit distinct binding affinities to sRNAs and show distinct roles in particular strain [12–15].

*P. fluorescens* 2P24, a rhizospheric bacterium originally isolated from the take-all decline soil, has been investigated for its ability to produce the secondary metabolite 2,4-diacetylphloroglucinol (2,4-DAPG), which contributes to the protection of various crop plants against soil borne disease caused by many plant pathogens [16]. The biosynthetic pathway of 2,4-DAPG has been clarified in several *Pseudomonas* strains. The 2,4-DAPG locus includes the four biosynthetic genes *phlACBD* that are transcribed as a single operon and is directly involved in the catalytic process of 2,4-DAPG production [17]. The first step in 2,4-DAPG biosynthesis is the formation of phloroglucinol (PG) from three units of malonyl-coenzyme A (malonyl-CoA) by the type III polyketide synthase PhlD [18]. The products of *phlACB* genes are together required for the transacetylation of PG to monoacetylphloroglucinol (MAPG) and then 2,4-DAPG [19].

Since the high concentration of 2,4-DAPG is toxic to the producing bacterium, biosynthesis of 2,4-DAPG is subtly modulated by complex regulatory networks in response to abiotic and biotic factors, and cell physiological status [20, 21]. The *phlF* and *phlH* genes, code for the pathway-specific transcriptional regulators of the production of 2,4-DAPG [19, 22]. Besides PhlF and PhlH, the Gac/Rsm cascade pathway plays a critical role in the production of 2,4-DAPG [1]. In *P. fluorescens* 2P24, the RsmA and RsmE proteins directly repress the translation of *phlACBD* mRNA, whereas four sRNAs RsmX, RsmX1, RsmY, and RsmZ derepress the translation of *phlACBD* mRNA by sequestering the RsmA and RsmE proteins, thereby inducing the production of 2,4-DAPG [15].

In this study, we found that RsmA and RsmE proteins contribute to bacterial growth advantages in *P. fluorescens* 2P24. Deletion of both of *rsmA* and *rsmE* genes could impair growth rate and cell density, whereas the growth rate and cell density was partially restored in the *rsmA rsmE phlD* triple mutant compared with that of the wild-type strain, suggesting that high levels of 2,4-DAPG in the cells could influence the growth of the *rsmA rsmE* double mutant. In addition, we demonstrated the role of the RsmA family proteins on type six secretion system (T6SS), swimming motility, and biofilm formation in *P. fluorescens*.

## Results

### High concentration of 2,4-DAPG in the cells impaired the growth of the *rsmA rsmE* double mutant

**Fig. 1:**
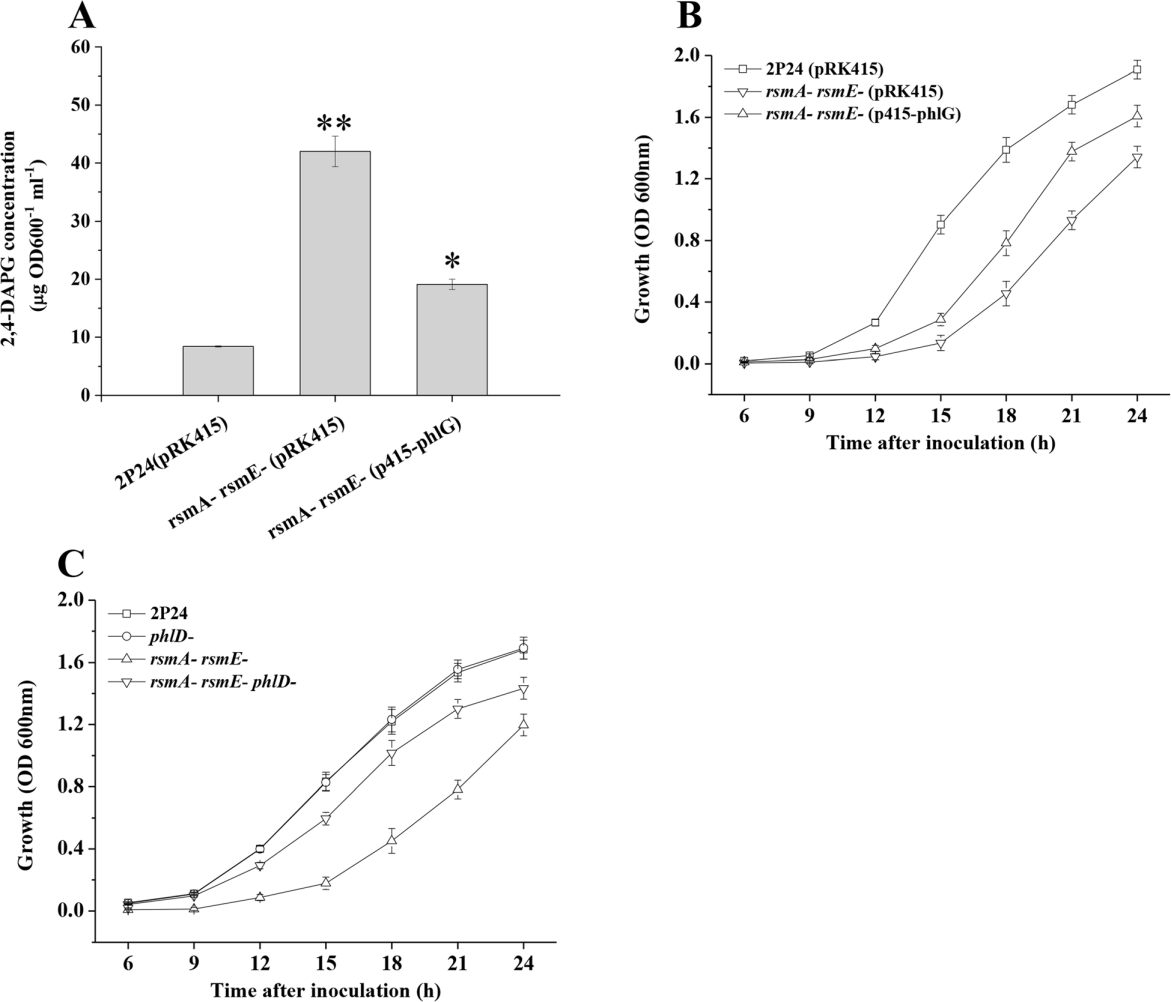
Overproduction of 2,4-DAPG influenced the growth curves of *P. fluorescens*. HPLC analysis of 2,4-DAPG production by strain 2P24 and its derivatives (**a**). Overexpression of *phlG* gene in the *rsmA rsmE* double mutant partially restored the growth of *P. fluorescens* (**b**). Deletion of *phlD* in the *rsmA rsmE* double mutant significantly influenced the growth of *P. fluorescens* (**c**). All experiments were performed in triplicate, and the mean values ± standard deviations are indicated. * indicates *P* < 0.05, and ** indicates *P* < 0.01

Previous study showed that the growth of the *rsmA rsmE* double mutant was severely impaired compared with the wild-type strain 2P24. Since high levels of 2,4-DAPG was toxic to the producing bacterium [20], to assess if the growth of the *rsmA rsmE* double mutant was impaired by the overdose of 2,4-DAPG in the cells, we overexpressed the *phlG* gene which encoding the 2,4-DAPG hydrolase and then measured the growth of 2P24 and its derivatives. As expected, introduction of the *phlG* gene cloned in the pRK415 plasmid (p415-phlG), into the *rsmA rsmE* double mutant, resulted in repression of 2,4-DAPG production (Fig. 1a). The growth rate of the *rsmA rsmE* double mutant with p415-phlG could be partially restored to that of the wild-type strain 2P24 (Fig. 1b). Furthermore, by deleting the *phlD* gene in the *rsmA rsmE* double mutant, the growth curve of the *rsmA rsmE phlD* triple mutant was significantly improved compared to that of the *rsmA rsmE* double mutant although was slightly lower compared to the wild-type strain 2P24 or the *phlD* mutant (Fig. 1c). Thus, these data suggested that overproduction of 2,4-DAPG contributes to the reduced growth of the *rsmA rsmE* double mutant.

### RNA sequencing reveals the *P. fluorescens* RsmA and RsmE regulon

To insight into the role of RsmA and RsmE in *P. fluorescens*, RNA sequencing (RNA-seq) was performed to define the RsmA and RsmE regulon of *P. fluorescens*. The genes that are significantly downregulated or upregulated are summarized in Table S1A & S1B. We defined the genes that showed a > 2-fold change of expression as differentially expressed genes (DEGs). In the *rsmA rsmE* double mutant, 621 genes were upregulated and 304 genes were downregulated compared to the wild-type strain (Table S1A & S1B).

**Fig. 2:**
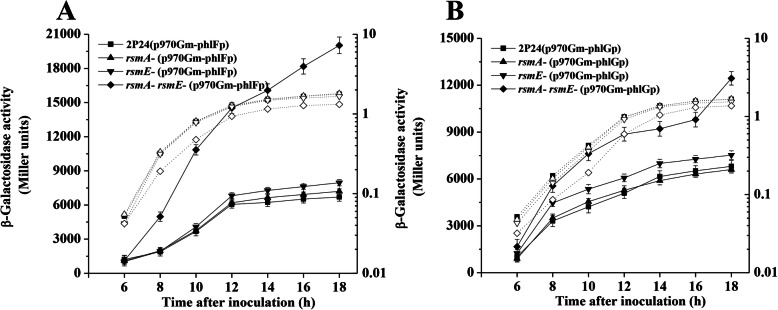
RsmA and RsmE regulated the expression of *phlF* and *phlG genes* in *P. fluorescens* 2P24. The transcriptional fusion *phlF*-*lacZ* (**a**) and *phlG*-*lacZ* (**b**) were measured in strain 2P24 and its derivatives. All experiments were performed in triplicate, and the mean values ± standard deviations are indicated. Growth is indicated by the dotted line

Based on the RNA-seq results, we observed that the expression of genes in *phl* operon (*phlACBD*) was increased by 145 to 587-fold (Table S1). To verify the complex regulatory role of *rsmA* and *rsmE* on the production of 2,4-DAPG, we measured the expression of *phlG* and *phlF* in the *rsmA rsmE* double mutant, respectively. Transcriptional fusion assays showed that both of the expression of *phlG* and *phlF* were significantly increased in the *rsmA rsmE* double mutant compared with that in the wild-type strain (Fig. 2). These results indicated a sophisticated role for the RsmA family proteins RsmA and RsmE in the production of 2,4-DAPG in *P. fluorescens*.

Among the genes upregulated in the *rsmA rsmE* double mutant, 21 encoded proteins that are associate with type six secretion system (T6SS), which is known as an important mechanism in interactions and pathogenesis against bacterial and eukaryotic cells. In addition, the RNA-seq data revealed that a significant number of genes influenced by RsmA and RsmE were involved in fatty acid metabolism (*fadA*, *fadB*, *fabG*, *fadH*, *psrA*), energy and carbon metabolism (*glpD*, *zwf*, *fahA*, *gcd*, *gltK*), and cell motility (*flaG*, *fliT*, *fliS*, *motA*, *motC*, *flgE*). Collectively, our data suggested that RsmA and RsmE are the pleiotropic regulators of secondary metabolism, cell motility, and other physiological processes.

### RsmA and RsmE negatively regulated the type six secretion system (T6SS)

**Fig. 3:**
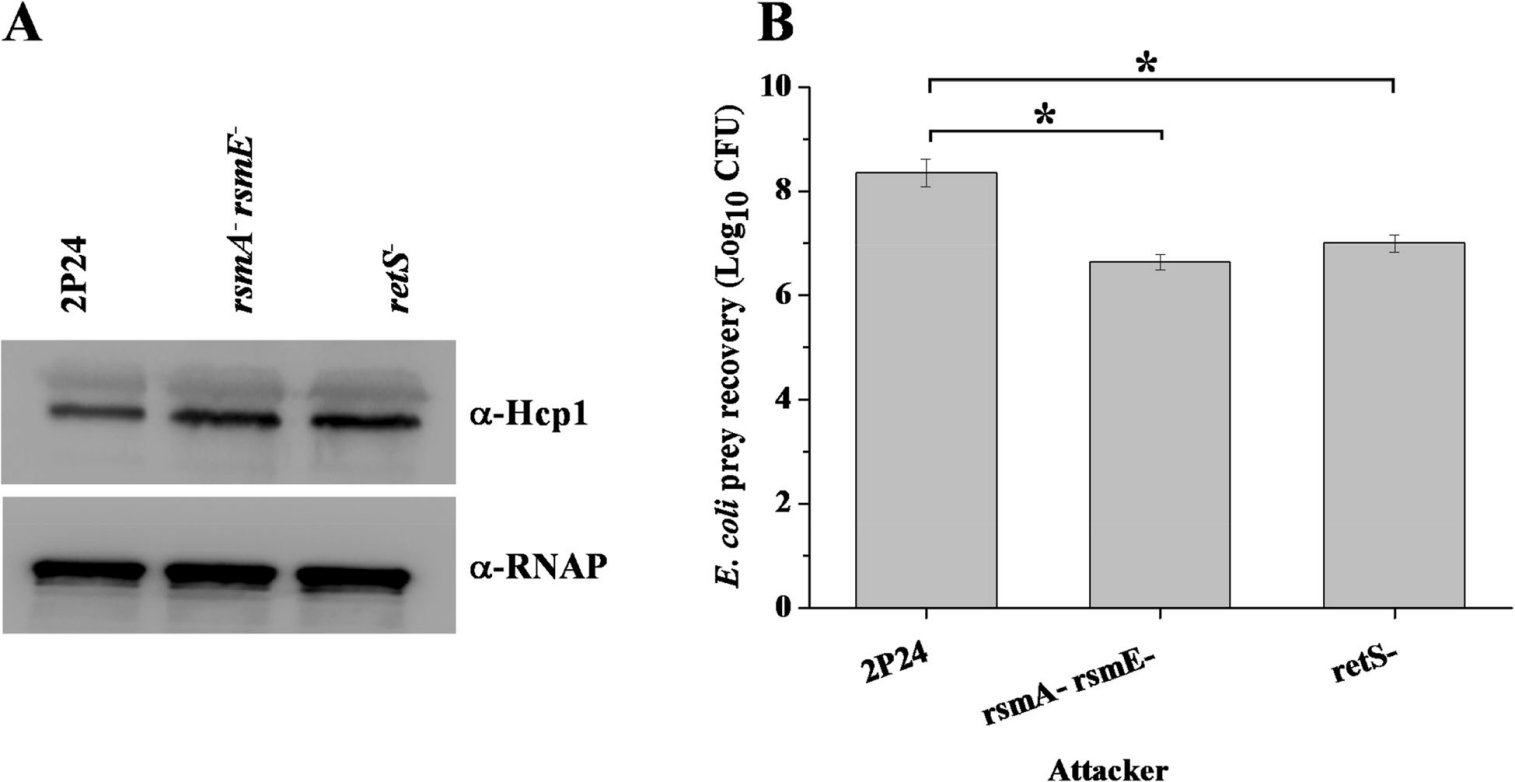
RsmA and RsmE repressed the T6SS in *P. fluorescens* 2P24. **a** Western blot analysis of Hcp1 protein level in wild-type strain 2P24 and its derivatives. **b** Quantification of bacterial killing assay after coincubation of *E. coli*, and various 2P24 attackers. All experiments were performed in triplicate, and the mean values ± standard deviations are indicated. * indicates *P* < 0.05

Bacterial T6SS plays an important role in both virulence and inter-bacterial competition and provide advantages to T6SS active strains in polymicrobial habitats [23]. Since many genes related to T6SS were up-regulated in the *rsmA rsmE* double mutant, we assayed the effect of the RsmA family protein on the production of T6SS structure protein Hcp1. Consistent with the RNA-seq data in the *rsmA rsmE* double mutant, Western blot analysis showed that this mutant produced higher amount of the Hcp1 protein than wild-type strain *P. fluorescens* 2P24 (Fig. 3a). Previously studies showed that the T6SSs could inject the T6SS toxins into bacterial preys [24]. We then performed the antibacterial assays using *E. coli* carrying the plasmid pHSG299 as prey and *P. fluorescens* 2P24 or its derivatives as predators. Similar to the *retS* mutant which triggered the T6SS [25], The *rsmA rsmE* double mutant caused a significant increase in survival of *E. coli* (Fig. 3b). Taken together, our results indicate that RsmA and RsmE repress the T6SS activity and play an important role for the inter-bacterial competition.

### The effect of RsmA and RsmE on cell motility and biofilm formation

**Fig. 4:**
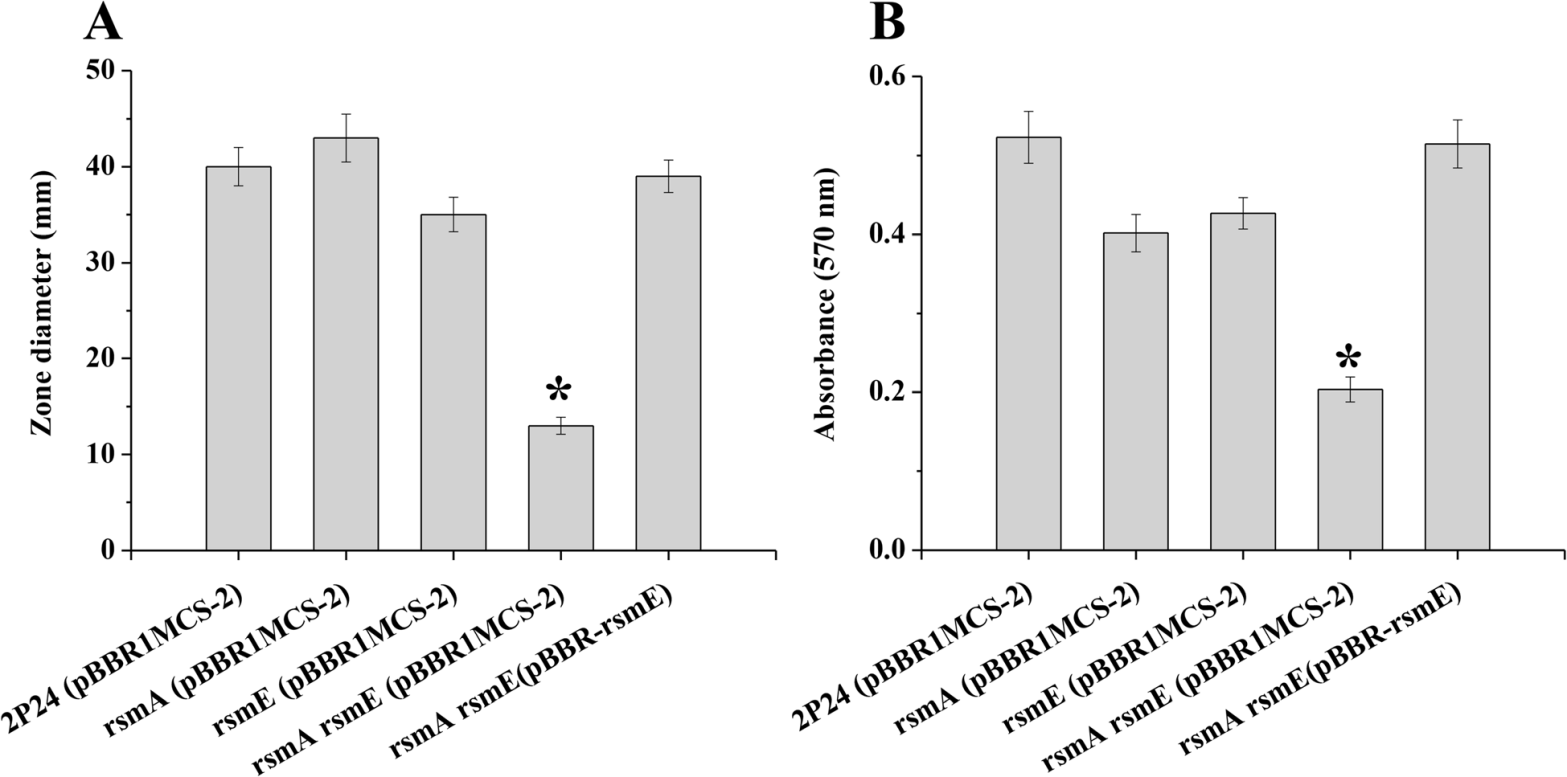
RsmA and RsmE controlled swimming motility and biofilm formation. **a** Swimming motility was tested on LB plates containing 0.3% agar. **b** Biofilm formation phenotype of wild-type strain 2P24 and its derivatives in LB medium. All experiments were performed in triplicate, and the mean values ± standard deviations are indicated. * indicates *P* < 0.05

Analysis of the *rsmA rsmE* double mutant RNA-seq data showed that expression of 20 genes involved in flagella biosynthesis and assembly was significantly changed, indicated a decrease in cell motility. To confirm this result, the motility of strain 2P24 and its derivatives was measured. Compared to the wild-type strain, the swimming motility was reduced by 72% in the *rsmA rsmE* double mutant (Fig. 4a). Motility is crucial in cell-to-cell adherence and attachment in early biofilm development. Whereas our data revealed a positive influence of the RsmA family proteins on biofilm formation (Fig. 4b). All observed phenotypes in the *rsmA rsmE* double mutant could be partly complemented by introducing the plasmid pBBR-rsmE (Fig. 4). Taken together, these results demonstrated that RsmA and RsmE are crucial for cell motility and biofilm formation in strain 2P24.

## Discussion

Plant growth-promoting rhizobacteria could antagonize plant pathogenic fungi by the production of antimicrobial secondary metabolites, such as 2,4-DAPG. Although 2,4-DAPG has antifungal, antibacterial, anthelminthic, and phytotoxic properties, it has toxicity to the producing bacterium at high concentrations. Many regulatory elements, including Gcd [26], Hfq [27], PsrA [28], RopS [29], and PhlG which directly or indirectly influence 2,4-DAPG biosynthesis, were regulated by the RsmA and RsmE (Table S1). The RsmA and RsmE proteins play an important role in regulating the production of antibiotic compound 2,4-DAPG, which is necessary for its biocontrol traits in *P. fluorescens* 2P24 [15]. In the present study, we revealed that regulation of the production of 2,4-DAPG by the RsmA family proteins RsmA and RsmE contributes to bacterial growth advantages.

The RsmA and RsmE proteins play a critical role for the production of 2,4-DAPG [1]. In *P. fluorescens* 2P24, RsmA and RsmE inhibited the expression of *phlACBD* at the translation level [15]. Varying numbers of RsmA orthologs have been identified in different *Pseudomonas* sp. [12, 14, 15]. The defects in the growth of the *rsm* mutants could be found in *P. putida* and *P. syringae* pv. *tomato* [12, 30]. Although no evidence showed that deletion of both of *rsmA* and *rsmE* genes influenced the growth of *P. protegens* CHA0 which could produce several antibiotics, including 2,4-DAPG [31], our data showed that the defect of bacterial growth was observed in the *rsmA rsmE* double mutant when compared with the wild-type strain 2P24 [15]. Interestingly, overexpression of *phlG* gene or deletion of *phlD* gene in the *rsmA rsmE* double mutant partly restored the growth curve of strain 2P24 (Fig. 1). Transcriptional fusion assays further suggested that the expression of *phlG* and *phlF* genes were negatively regulated by RsmA and RsmE (Fig. 2), indicating that RsmA and RsmE could balance the concentration of 2,4-DAPG in the cells by fine-tuning the role of multiple regulators of the production of 2,4-DAPG. The biosynthesis of 2,4-DAPG leads to lower bacterial growth rates due to the increased metabolic costs in the cells [22]. Deletion of *phlD* gene in the *rsmA rsmE* double mutant could not completely restore the growth curve of strain 2P24 (Fig. 1), whereas the deficiency of the growth could be restored when the *rsmA rsmE* double mutant was growth in minimal medium with glucose as a carbon source [15], indicating that RsmA and RsmE play an important role in central carbohydrate metabolism. Our RNA-seq analysis supported this hypothesis which identified several genes that are known to be involved in fatty acid metabolism, carbon metabolism, and pleiotropic molecule cyclic diguanylate production (Table S1). In addition, our data suggested that RsmA and RsmE are the global regulators in a complex regulatory network that provides advantages to strain 2P24 in polymicrobial environments. For instance, inactivation of both of *rsmA* and *rsmE* increased the levels of Hcp1 and provided a growth advantage against *E. coli* in *P. fluoresces* 2P24 (Fig. 3). These results were in agreement with the previously proposed role of *rsmA* in affecting *tssA1*, *tse6*, and *tsi4* [24]. Together, our data suggested that RsmA and RsmE might play a critical role in fine-tuning the concentration of 2,4-DAPG to maintain the metabolic homeostasis and improve the competitive advantages of 2P24 against other microbes living in or nearby the rhizosphere.

We also identified 20 genes involved in flagella biosynthesis and assembly by genome-wide expression analysis (Table S1B). According to these observations, mutation in the *rsmA* and *rsmE* genes resulted in decreasing motility (Fig. 4a). Bacterial motility and biofilm formation are inversely regulated in Gram-negative bacteria [32]. Interestingly, the results of this work strongly suggest that RsmA and RsmE positively affected biofilm formation (Fig. 4b). Previous study showed that bacterial secondary messenger cyclic diguanylate (c-di-GMP) influences biofilm development [33]. Many genes involved in c-di-GMP turn over were regulated by RsmA and RsmE (Table S1). We speculate that RsmA and RsmE may interact with the c-di-GMP signaling pathway to regulate biofilm formation in *P. fluorescens*.

In summary, these results demonstrated that several important intracellular activities and behaviors were regulated by the RsmA family proteins RsmA and RsmE in plant-associated *P. fluorescens* 2P24. By fine-tuning the concentration of 2,4-DAPG and carbon metabolism in the cells, RsmA and RsmE could contribute to growth advantages of strain 2P24.

## Conclusions

The plant-associated *P. fluorescens* 2P24 can colonize root of many crops and protect them from infection by phytopathogens. In this study, our data showed that the regulation of 2,4-DAPG by RsmA and RsmE was complicated and involved in many specific elements. In addition, several important intracellular activities and behaviors, such as growth curve, carbon metabolism, T6SS, the formation of biofilm, and motility were regulated by RsmA and RsmE in strain 2P24. These findings provide a new understanding of the regulatory role of RsmA and RsmE in *P. fluorescens*.

## Methods

### Bacterial strains, plasmids, and growth conditions

The bacterial strains and plasmids used in this study are listed in Table 1. *Escherichia coli* was routinely grown in lysogeny broth (LB) medium at 37 °C. Unless otherwise indicated, *P. fluorescens* 2P24 and its derivatives were grown in LB medium, King’s B medium (KB) [37], or ABM medium [38] at 28 °C. The concentration of antibiotics was added as follows: ampicillin (50 μg/ml), kanamycin (50 μg/ml), tetracycline (20 μg/ml).

**Table 1:** bacterial strains, plasmids, and oligonucletoides used in this study

Strains, plasmids or oligonucletoide	Relevant characteristics*	Reference or source
Strains		
*Pseudomonas fluorescens*		
2P24	Wild-type, Ap^r^	[16]
PM206	In-frame deletion of *retS*, Ap^r^	[34]
WPM30	In-frame deletion of *phlD*, Ap^r^	This work
WPM12	Double deletion of *rsmA* and *rsmE*, Ap^r^	[15]
WPM31	Triple deletion of *rsmA*, *rsmE*, and *phlD*, Ap^r^	This work
*E. coli* DH5α	*supE44 lacU*169 (*ϕ80lacZ* M15) *hsdR*17 *recA*1 *endA*1 *gyrA*96 *thi*-1 *relA*1	[35]
Plasmids		
p2P24Km	Sucrose-based counter-selectable plasmid, Km^r^	[36]
p2P24Km-phlD	Plasmid p2P24Km carrying a deleted *phlD* gene, Km^r^	This work
p970Km-phlFp	*phlF*-*lacZ* transcriptional fusion, Km^r^	This work
p970Km-phlGp	*phlG*-*lacZ* transcriptional fusion, Km^r^	This work
p415-phlG	pRK415 containing the *phlG* gene, Tet^r^	[22]
pHSG299	Cloning vector, Km^r^	TaKaRa
Oligonucletoides	Sequence (5′-′3) ^a^	Comment
phlD-F1-EcoRI	AAGAATTCATGGCGATGGTGCGCCT	*phlD* null mutant construction
phlD-R1–680	GAATTTTCCGTCCGCCTGTATGGAACATGAAACCCGTGCACGATGTCACA	
phlD-F2–700	TGTGACATCGTGCACGGGTTTCATGTTCCATACAGGCCGGACGGAAAATTC	
phlD-R2-SalI	AAGTCGACCAGGCTGGTGATCAATG	
phlG-PFBamHI	TAGGATCCAGTTGCA CCAACCGAGC	*phlG-lacZ* transcriptional fusion
phlG-PRBamHI	ATGGATCCGGCACGCTGATCTTCGAGC	
phlF-PFBamHI	ACGGATCCAGATCTTAAGGGTTTCTAT	*phlF*-*lacZ* transcriptional fusion
phlF-PRBamHI	GTGGATCCATAAGGATTGGTGCAG	

^*^Ap, ampicillin; Km, kanamycin; Tet, tetracycline

^a^Restriction site inserted in the primer for the cloning strategy are underlined

### Mutant construction

The *phlD* in-frame deletion mutant was constructed by allelic exchange mutagenesis [36]. Briefly, upstream and downstream fragments flanking the *phlD* gene were amplified by PCR using primers phlD-F1-EcoRI/phlD-R1–680 and phlD-F2–700/phlD-R2-SalI, respectively. The upstream and downstream PCR products were used as templates in fusion PCR with primers phlD-F1-EcoRI/phlD-R2-SalI. Subsequently, the fusing fragment was cloned into the suicide vector p2P24Km [36]. The resulting plasmid p2P24-phlD was introduced into strain 2P24 and the *rsmA rsmE* double mutant by electroporation and double-crossover recombination events were selected [39]. Substitution was confirmed by PCR and sequencing.

### RNA-seq analysis

To test the effect of the *rsmA* and *rsmE* genes on the transcriptome in *P. fluorescens* 2P24, cells were cultured to early stationary phase (OD_600_ = 1.0) in LB medium. Total RNA was conducted using the RNeasy minikit (Qiagen, MD, U.S.A.). The Ambion Turbo DNA-free kit was applied to remove contaminant DNA. After removal of rRNA by using the Ribo-Zero rRNA removal kit (Illumina, CA, U.S.A.), mRNA was used to generate the cDNA library according to NEBNext UltraTM II RNA Library Prep Kit, which was then sequenced using an Illumina HiSeq 2500 platform. High-quality reads were aligned to the *P. fluorescens* 2P24 genome (GenBank accession no. CP025542). From the resulting alignments, SAMtools version 1.6 [40] was applied to sort the bam file. The differentially expressed genes were identified by performing Cuffdiff version 2.2.1 [41] with a *p* value smaller than 1e-5. Each sample in the RNA-seq was repeated three times.

### Construction of the transcriptional *lacZ* fusion and measurements of β-galactosidase activity

To construct the *phlF*-*lacZ* and *phlG*-*lacZ* transcriptional fusions, a 700-bp DNA fragment upstream of *phlF* and a 540-bp fragment upstream of *phlG* were cloned separately into pRG970Km [24], ahead of a promoterless *lacZ* gene, to gain p970Km-phlFp and p970Km-phlGp, respectively. The primers used are listed in Table 1.

Strains carry the *lacZ* transcriptional fusions were grown in LB medium with agitation at 28 °C. The bacterial cells were collected by centrifugation and the β-galactosidase activity was measured using the method as reported previously [42].

### Quantification of 2,4-DAPG

Strain 2P24 and its derivatives were grown in 20 ml KBG (KB broth supplemented with 2% glucose) at 28 °C for 48 h. 2,4-DAPG was extracted from culture supernatant and quantitatively analyzed according to the method described previously [43].

### Swimming motility assay

Overnight LB culture was inoculated, and transferred at 1: 1000 to 2 ml fresh LB medium, and then grown at 28 °C until it reached an OD600 of 0.8. Two microliters of the cultures were spotted on soft LB plates (0.3% agar). The plates were inoculated at 28 °C for 24 h.

### Biofilm formation assay

A biofilm detection experiment was performed as reported previously [44]. In brief, overnight bacterial culture was transferred to a 2-ml EP tube containing 1 ml LB medium at an OD_600_ of 0.5 and cultured statically at 28 °C for 2 d. Crystal violet (0.1%) was used to stain biofilm adhered to the tubes for 15 min. The tubes were washed gently three times with ddH_2_O, and the remaining crystal violet was fully dissolved in 200 μl of 95% ethanol and the absorbance was detected at 570 nm.

### Western blot analysis

*P. fluorescens* cells were grown in LB at 28 °C for 12 h and 1-ml samples were taken. Cells were then collected by centrifugation, re-suspended in PBS buffer, and lysed by sonication. The protein in crude lysates was quantified using the Bradford protein assay (Bio-Rad, CA, U.S.A.). Samples were boiled before being loaded onto 12% sodium dodecyl sulfate polyacrylamide (SDS-PAGE) gels. Proteins were then transferred onto a polyvinylidene fluoride membrane (PVDF) (Millipore, MA, U.S.A.). Blots were washed with PBS containing 0.05% Tween-20 and probed with an anti-Hcp1 antibody (1:2000). Anti-RNA polymerase monoclonal antibody (1:2000) (Neoclone, WI, U.S.A.) was used as a control. Signals were then developed using the chemiluminescence detection kit (Thermo Fisher, MA, U.S.A.).

### T6SS competition assays

Competition assays were performed as previously described [45]. Overnight bacterial cultures were adjusted to OD_600_ of 1 in PBS solution and mixed in a 1:1 ratio [*P. fluorescens*-*E. coli* (pHSG299) as prey]. Bacteria were spotted on LB agar plates to co-culture at 28 °C for 5 h. The competition was then quantified by counting colony-forming units on antibiotic selection.

### Statistical analysis

Data were tested for normality and analyzed using unpaired Student’s *t* test. Asterisks indicated *P* values (^*^, *P* < 0.05; ^**^, *P* < 0.01), and results were presented as the mean standard deviation. Each experiment was performed three times in similar conditions.

## Supplementary information

**Additional file 1: Table S1.** The genes regulated by the RsmA and RsmE proteins

**Additional file 2: Figure S3.** a A Western blot analysis of Hcp1 protein level in 2P24 (lane 1), the *rsmA rsmE* double mutant (lane 2), and the *retS* mutant (lane 3). (from left to right: lane 1 to 3). Figure S3 b Western blot analysis of RNA polymerase beta protein level (as a loading control) in 2P24 (lane 1), the *rsmA rsmE* double mutant (lane 2), and the *retS* mutant (lane 3). (from left to right: lane 1 to 3)

## Data Availability

The genome sequence of *Pseudomonas fluorescens* 2P24 has been submitted to GenBank with accession number CP025542. The datasets used and/or analyzed during this study available from the corresponding author on reasonable request.

## Abbreviations

2,4-DAPG: 2,4-diacetylphloroglucinol
sRNA: small non-coding RNA
UAS: upstream activating sequence
PG: phlotoglucinol
MAPG: monoacetylphloroglucinol
T6SS: type six secretion system
RNA-seq: RNA sequencing
DEGs: differentially expressed genes
c-di-GMP: cyclic diguanylate
