# Supplementary material for: Pleiotropic effects of RsmA and RsmE proteins in Pseudomonas fluorescens 2P24
Source: BMC Microbiol. 2020 Jul 2;20:191. doi: 10.1186/s12866-020-01880-x (PMC7331252; doi:10.1186/s12866-020-01880-x)
Supplement: Supplementary file 2 — Additional file 2: Figure S3. a A Western blot analysis of Hcp1 protein level in 2P24 (lane 1), the rsmA rsmE double mutant (lane 2), and the retS mutant (lane 3). (from left to right: lane 1 to 3). Figure S3 b Western blot analysis of RNA polymerase beta protein level (as a loading control) in 2P24 (lane 1), the rsmA rsmE double mutant (lane 2), and the retS mutant (lane 3). (from left to right: lane 1 to 3) [file 12866_2020_1880_MOESM2_ESM.docx]

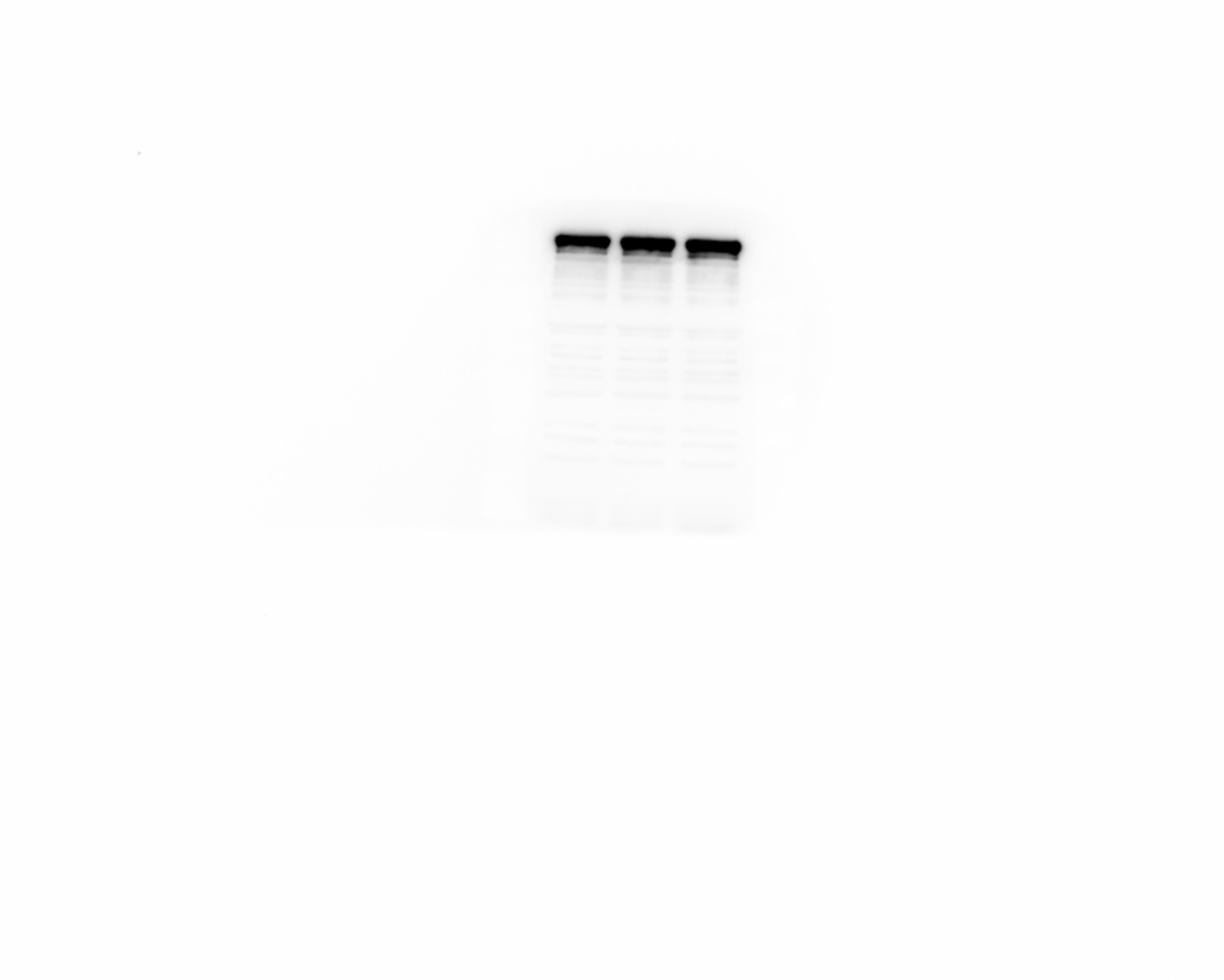

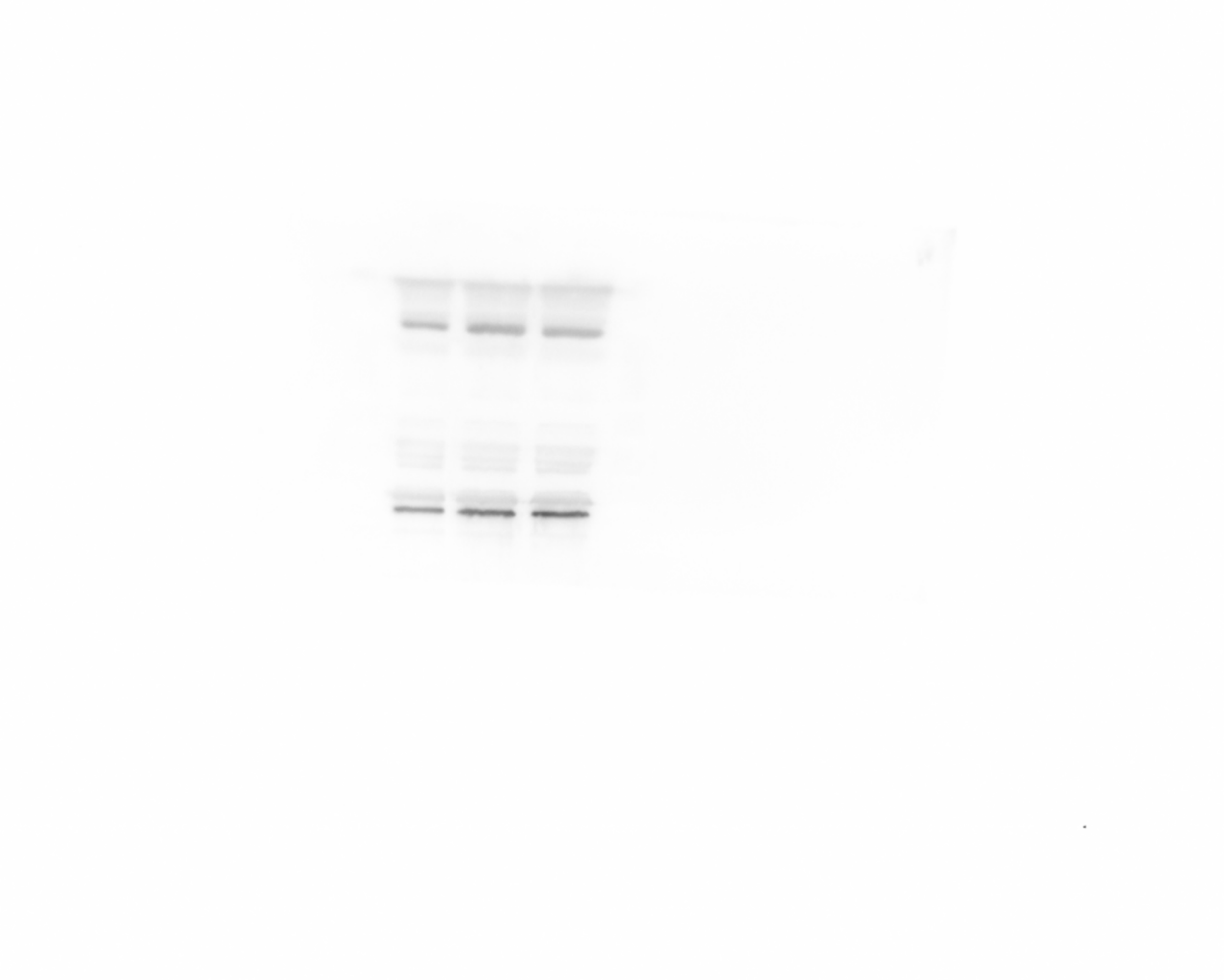
Fig. 3a Western blot analysis of Hcp1 protein level in 2P24 (lane 1), the *rsmA rsmE* double mutant (lane 2), and the *retS* mutant (lane 3). (from left to right: lane 1 to 3).

Fig. 3b Western blot analysis of RNA polymerase beta protein level (as a loading control) in 2P24 (lane 1), the *rsmA rsmE* double mutant (lane 2), and the *retS* mutant (lane 3). (from left to right: lane 1 to 3)

------------------------------------------------

Different edit of the images.

Fig. 3a Western blot analysis of Hcp1 protein level in 2P24 (lane 1), the *rsmA rsmE* double mutant (lane 2), and the *retS* mutant (lane 3).

Fig. 3b Western blot analysis of RNA polymerase beta protein level (as a loading control) in 2P24 (lane 1), the *rsmA rsmE* double mutant (lane 2), and the *retS* mutant (lane 3).
